# Patterns and Predictors of Adherence to Statin Therapy Among Older Patients: Protocol for a Systematic Review

**DOI:** 10.2196/resprot.7195

**Published:** 2017-03-07

**Authors:** Richard Ofori-Asenso, Ella Zomer, Andrea Curtis, Andrew Tonkin, Mark Nelson, Manoj Gambhir, Danny Liew, Sophia Zoungas

**Affiliations:** ^1^ Department of Epidemiology and Preventive Medicine Monash University Melbourne Australia; ^2^ Discipline of General Practice University of Tasmania Hobart Australia

**Keywords:** statin therapy, adherence, compliance, persistence, elderly patients, geriatrics

## Abstract

**Background:**

The benefits of statin therapy are significantly compromised by noncompliance. Although elderly patients may have particular challenges with medication adherence and persistence, previous reviews on statin adherence have not focused on this population. Additionally, comparisons of adherence and persistence specific to statin indication (primary or secondary prevention) have not been thoroughly explored.

**Objective:**

We aim to assess the extent of, and factors associated with, adherence and persistence to statin therapy among older populations (aged ≥65 years).

**Methods:**

A systematic review will be undertaken according to the Preferred Reporting Items for Systematic Reviews and Meta-Analyses recommendations. Searches will be performed using multiple electronic databases (Ovid MEDLINE, EMBASE, PsycINFO, CINAHL, Cochrane Central Register of Controlled Trials, Database of Abstracts of Reviews of Effects, and the National Health Service Economic Evaluation Database) to identify relevant randomized trials and observational studies that evaluated statin adherence and/or persistence as an outcome. Eligible studies will include those involving community-living or outpatient elderly individuals. The methodological quality of randomized controlled trials (RCTs) will be assessed via the Joanna Briggs Institute’s critical appraisal checklist for RCTs, and the quality assessment of observational studies will be undertaken using a set of questions formulated with resort to the National Institute of Health Quality Assessment Tool for Observational Cohort and Cross-Sectional Studies. When possible, meta-analyses will be conducted using random-effect modeling and generic inverse variance analyses for adjusted-effect estimates. Heterogeneity across studies will be quantified using the I^2^ statistic. The presence of publication bias will be assessed using funnel plots and Egger’s regression tests. A leave-one-out sensitivity analysis will also be conducted to assess the impact of individual study results on pooled estimates. To explore possible sources of heterogeneity across studies, subgroup analyses will be performed based on covariates such as study design, statin indication, country of study, and length of patient follow-up.

**Results:**

The electronic database searches were completed in December 2016. Retrieved articles are currently being screened and the entire study is expected to be completed by June 2017.

**Conclusions:**

This systematic review will provide further understanding of the patterns of, and barriers to, statin adherence and persistence among older patients. The findings will inform clinical practice and the design of appropriate interventions.

**Trial Registration:**

PROSPERO CRD42016053191

## Introduction

Beginning with their discovery in the 1970s, and becoming available for clinical use in the 1980s [1], 3- *hydroxymethyl*-3-methylglutaryl coenzyme A (HMG-CoA) reductase inhibitors (statins) are one of the most frequently prescribed medications, with global users estimated to be more than 1 billion in 2014 [2]. Several clinical trials and reviews have reported statins to be highly efficacious for the prevention of cardiovascular events [3,4]. The biological role of statins in the reduction of cholesterol levels has also led to suggestions of possible use of statins as preventive agents for other conditions, such as dementia [5] and cancer prevention [6,7].

Statins are generally well tolerated by most patients [8], but nonadherence has been reported across observational studies and from analyses of large population-based registries [9]. A meta-analysis estimated that only approximately half (49%) of all patients in observational studies were adherent to statin therapy at 1 year of follow-up [10], although much higher adherence (90.3%) has been observed in randomized trials [10], in which participants were often motivated, and rigorous follow-up was usually in place.

Furthermore, studies have demonstrated increased risk of adverse outcomes following poor statin adherence [11-13]. In some cases, outcomes among nonadherers and those who discontinued statin therapy were found to be even worse than for those who had not initiated treatment [13].

Several systematic reviews have been published on adherence among statin users [9,14,15]. However, no reviews have focused on elderly patients who may face unique challenges with adherence and persistence, especially since this group experiences greater comorbidity and polypharmacy, which are two key contributing factors to these phenomona [16-18]. Additionally, the balance of the risks and benefits associated with statin therapy (particularly for primary prevention) remains unclear and highly debated for the elderly [19,20]. These factors may further impact on patients’ willingness to adhere to treatment.

Ongoing demographic changes, characterized by an increasing number of elderly individuals [21], suggests that this population will constitute a significant proportion of current and future statin users. In light of this reality, a systematic review that seeks to explore issues of adherence and persistence specific to the older population is necessary to: (1) identify relevant barriers, (2) compare the level of adherence and persistence relative to statin indication (primary or secondary prevention), and (3) inform the design of appropriate interventions.

## Methods

This systematic review will be carried out in line with recommendations specified in the Preferred Reporting Items for Systematic Reviews and Meta-Analyses (PRISMA) statement [22]. The protocol has also been prepared in accordance with PRISMA Protocols guidelines (Supplemental File 1) [23].

### Review Objectives

This study will assess the extent of, and factors associated with, adherence and persistence to statin therapy among older populations. More specifically, the review objectives are:

- To quantify the proportion of older statin users who are adherent, and compare rates reported in randomized trials to those reported in observational studies, as well as by statin indication (primary vs secondary prevention).
- To assess the level of persistence to statin therapy among older patients, and compare rates between primary and secondary prevention patients.
- To assess, summarize, and provide estimates of risk indicators associated with nonadherence and nonpersistence to statin therapy in the elderly.

The key components of the review will follow the standard *population, intervention, comparison, outcome, and study design* (PICOS) approach (Textbox 1) [24].

**Key components of the systematic review following the standard PICOS approach.**

- Population: older patients (aged ≥65 years) undergoing primary or secondary prevention treatment
- Intervention: all statins (HMG-CoA reductase inhibitors)
- Comparison: none
- Outcomes: proportion of patients who were adherent, proportion of patients who were persistent at predefined periods, risk factors associated with nonadherence, risk factors associated with nonpersistence
- Study design: randomized controlled trials and observational studies (prospective and retrospective)

### Intervention

The study will focus on all statins and will consider their use for primary and secondary prevention among elderly populations. The outcome(s) evaluated will not be compared across the different statins, and comparisons related to dosing regimen (eg, once or twice daily dosing) will not be carried out.

### Outcomes

The use or nonuse of prescribed medicines by patients is often described using a variety of terms such as *adherence*, *compliance*, *persistence*, or *concordance*, which can cause confusion [25-27]. Nonetheless, *adherence*, *compliance*, and *concordance* are often used in relation to instances involving medication doses that are missed [28], and *persistence* is often used in relation to the time from initiation to termination of treatment [29]. Medication concordance, conversely, is usually used to emphasize that the doctor and patient have achieved some level of agreement regarding the therapeutic goal(s) [30]. For this review, *adherence* and *compliance* will represent the same thing, and be used interchangeably. When reference is made to *persistence*, this will relate to the duration of statin use. The benefits of statin therapy are likely to accrue over time [31], making persistence an important measure that is closely related to adherence. Adherence and persistence are detailed below.

#### Adherence

Adherence refers to, “the extent to which a patient acts in accordance with the prescribed interval and dose of a dosing regimen” [29]. At the individual level, adherence may be estimated via the proportion of doses taken (PDT), which is calculated as: (number of pills taken in time Y)/ (number of pill prescribed for time Y)*100 [32]. Adherence may also be expressed as the proportion of days covered (PDC) which is calculated as: (total days drugs available)/(days in follow-up period)*100 [33]. Furthermore, adherence may be quantified in terms of the medicines possession ratio (MPR) which is calculated as: (number of days of medication supplied within refill interval)/(number of days in refill interval)*100] [33]. In this review, we will define adequate adherence to represent instances in which the MPR, PDC, and PDT are each ≥80%[10,14]. As such, for studies adopting the MPR, PDC, PDT, and similar methodologies, we will only include those that report sample-level adherence rates based on the application of the ≥80% threshold to individual patients. Studies that measure adherence as a continuous (rather than dichotomous) variable using MPR, PDC, PDT, and similar methodologies will be excluded. Studies that utilize other tools, including self-reported scales (eg, Morisky Medication Adherence Scale [34]), and classify patients as *adherent* will use the tool’s established recommendations. Of note, adherence rates in this review will not include *primary nonadherence*, which indicates instances that patients failed to fill their first statin prescription [35].

#### Persistence

Persistence refers to the continuous use of medication by patients for the required duration. Although various methods exist for estimating medication persistence, including the use of medication possession at a fixed point in time, the most commonly adopted approach involves quantifying the gaps between prescription refills [36]. Patients are often considered to have discontinued (been nonpersistent) if they have exceeded a permissible gap (number of days allowed between refills). No standardized permissible gap typically exists, as this will often be dictated by the length of previous prescriptions. However, a range of 1.5-3 times the days’ supply of preceding prescription has often been used [37]. We will consider all studies that report sample-level persistence rates, whether based on a defined permissible gap or where there is evidence of discontinuation, such as patient self-reports.

This study will also assess factors that are reported to influence adherence and persistence to statin therapy among elderly patients. These factors will be grouped under five themes, in line with the World Health Organization’s classification of predictive factors of nonadherence [38,39]: (1) patient-related factors (eg, gender), (2) socioeconomic factors (eg, educational status, family support), (3) therapy-related factors (eg, concurrent drug therapy, adverse effects), (4) health system-related factors (eg, proximity to pharmacy), and (5) disease-related factors (eg, presence of comorbidities).

### Study Inclusion and Exclusion Criteria

For the current review, both observational (prospective and retrospective) studies and randomized controlled trials (RCTs) that evaluated statin adherence and/or persistence as an outcome will be included. In line with similar reviews [28], we will focus on noninstitutionalized persons and will exclude studies conducted solely on participants within nursing or care homes and inpatient settings. Studies in which medications were administered by a carer or healthcare personnel will be excluded [28]. For studies to be eligible, adherence and/or persistence should also have been assessed over a defined period using an objective measure (eg, pill count, medication refill data) or via a validated self-reported instrument. Studies that mixed older (≥65 years years) and younger individuals (<65 years) will be excluded unless specific results have been presented for the elderly population, or where efforts to retrieve such data from authors have been successful. Studies that do not report adherence and/or persistence solely on statins (eg, where statins are mixed with other medications including other lipid-lowering drugs) will be excluded. Studies with sample sizes <50 will be excluded [40]. No country restrictions will be imposed.

### Search Strategy

To identify appropriate studies for the review, searches were performed using Ovid MEDLINE, EMBASE, CINAHL, PsycINFO, Database of Abstracts of Reviews of Effects, National Health Service Economic Evaluation Database, and the Cochrane Central Register of Controlled Trials. The main keywords that were used included, “statins *or* HMG-CoA reductase inhibitors *or* individual generic and propriety names” and, “medication compliance *or* adherence *or* persistence *or* treatment refusal *or* drop out *or* discontinuation”. Table 1 presents the search strategy for Ovid MEDLINE developed in consultation with an information management specialist (librarian) [41]. This search strategy was replicated for the remaining databases, and modifications were made in-line with individual database requirements if necessary. All electronic searches were completed on December 12, 2016 and only studies published before this date will be considered for inclusion in this review. The reference list of all selected articles will be screened for additional studies. In view of limited time and resources, only studies published in English will be considered for the review.

**Table 1:** Search strategy developed for Ovid MEDLINE.

*Block 1: Statins*	
	1. exp hydroxymethylglutaryl-coA reductase inhibitors/
	2. statin*.mp.
	3. ([hmg-coa reductase or hydroxymethylglutaryl coa or hydroxymethylglutaryl-coenzyme a] adj2 inhibit*).mp.
	4. (atorvastatin or lipitor).mp.
	5. (simvastatin or zocor).mp.
	6. (cerivastatin or lipobay or baychol).mp.
	7. (lovastatin or mevacor or altoprev).mp.
	8. (fluvastatin or lescol).mp.
	9. mevastatin.mp.
	10. (rosuvastatin or crestor).mp.
	11. (pitavastatin or livalo).mp.
	12. (pravastatin or pravachol).mp.
	13. ([lipid or cholesterol] adj3 lower*).mp.
	14. (antilipid* or anti-lipid*).mp.
	15. or/1-14
*Block 2: Adherence/compliance/persistence*	
	16. exp patient compliance/
	17. exp medication adherence/
	18. (complian* or noncomplian* or discontinu* or adher* or persist* or concordance or nonadher* or nonpersist* or dropout* or drop-out*).mp.
	19. (patient* adj3 [attitude* or acceptance* or satisf*]).mp.
	20. (treatment* adj3 [stop* or abandon* or refus*]).mp.
	21. or/16-20
*Block 3: Study designs*	
	22. ([observation* or prospective* or retrospective*] adj2 [study or studies]).mp.
	23. randomized controlled trial.pt.
	24. controlled clinical trial.pt.
	25. randomized.ab.
	26. placebo.ab.
	27. drug therapy.fs.
	28. trial.ab
	29. groups.ab.
	30. or/22-29
*Search hits*	
	31. 15 and 21 and 30
*Limits*	
	32. exp animals/not humans.sh.
	33. 31 not 32
	34. limit 33 to English language

### Study Selection

Results of individual database searches will be exported to Endnote referencing software and duplicates will be removed. Titles and abstracts of studies will initially be screened, and those that are likely to be of interest and relevance will be shortlisted for full-text examination. The full-text assessment will be undertaken with consideration of the study inclusion/exclusion criteria. We will link studies with multiple publications. The study-screening process will be conducted by RO and validated by another member of the team. Disagreements will involve consultation with a third team member and any issues will be addressed using a consensus-based approach. The entire screening and selection process will be summarized using a PRISMA flow chart and reasons for exclusion of studies will be documented.

### Study Quality Appraisal

The methodological quality of RCTs will be assessed using the Joanna Briggs Institute’s critical appraisal checklist for RCTs (Supplemental File 2) [42]. This tool includes 13 questions that relate to randomization, allocation concealment, blinding, and data analysis. For observational studies, quality assessments will be undertaken using a set of questions formulated with resort to the National Institutes of Health (NIH) Quality Assessment Tool for Observational Cohort and Cross-Sectional Studies (Supplemental File 3) [43]. The NIH tool includes 14 questions that relate to reporting, sample size estimation, loss-to-follow-up, outcome measurement, validity, and generalizability. The quality of each study will be rated as either *good*, *fair*, or *poor*.

### Data Extraction

A data extraction tool that incorporates relevant items (recommended by the Cochrane handbook for systematic reviews of interventions [44]) will be used to extract and record data from the studies. The descriptive characteristics of each study, including citation, author details, year, country, study design, sample size, participant composition (eg, percentage of females), and statin indication (primary or secondary prevention) will be summarized. Additionally, we will collect information on adherence and persistence definitions and measurement technique(s), as well as adherence and persistence rates and reported predictive factors. If adherence is measured using more than one technique, the average adherence will be calculated and the results of various techniques will be extracted for a sensitivity analysis. Studies evaluating the impact of an intervention will only have baseline (or comparative control group) results selected. We anticipate variations in the duration of patients’ follow-up, and we will report adherence and persistence rates for both short-term (eg, 3 months, 6 months, 12 months) and long-term (24 months or more, up to 5 years) follow-ups, to the extent that available data allows. Individual study data will be extracted separately by two members of the team (RO and another team member) and compared to ascertain consistency and reliability. Any discrepancies will be resolved through consensus-based discussions among the reviewers. Corresponding authors will be contacted via email for assistance if missing or unclear data cannot be reliably extracted. Authors that have not responded within a set period of time will be declared *unreachable*.

### Analyses

Adherence and persistence represent different aspects of medication usage [28,29], so separate analyses of these outcomes will be conducted. Adherence and persistence rates reported from individual studies will be logit transformed using the formula: *x*=logit (*p*)=In(*p* /1- *p*), where  *p* is the proportion of patients who were considered to be adherent or persistent. Meta-analyses will be performed using a random-effect model weighted by the inverse variance. Results will be back-transformed into proportion (using the formula *p*=logit^-1^[*x*]=e^x^*/* e^x^+1) to ensure comprehensive interpretation of results [45]. Cases in which adherence or persistence are reported only by distinct groups (ie, gender, age groupings) will have subgroups included as separate terms in the meta-analyses. For factors reported to be associated with adherence or persistence, the odds ratios (ORs) along with 95% CIs will be used for quantitative pooling. If the measures of association are reported as other parameters (eg, relative risk and standard mean difference), these will be converted to ORs. Instances in which a study reports an insignificant association without data will have an OR of 1 assigned. Log ORs and standard errors will be combined using the generic inverse-variance method. For each predictive factor, results will be quantitatively pooled, if reported by a minimum of two studies. Results of meta-analyses will be presented as forest plots. The level of heterogeneity resulting from variations of effects from individual studies will be assessed based on I^2^statistics. I^2^values of 30-60% may denote moderate heterogeneity, 50-90% substantial heterogeneity, and 75-100% considerable heterogeneity [46]. We will evaluate the presence of publication bias by assessing the asymmetry of effect sizes in funnel plots using the trim-and-fill method [47], and Egger’s regression tests will be used to quantify small study effects [48]. Additionally, a leave-one-out sensitivity analysis will be conducted by iteratively removing one study at a time to assess the impact of each study on the overall pooled adherence and persistence estimates [49]. Subgroup analyses will be performed based on covariates such as study design, country of origin, method used to estimate adherence, length of patient follow-up, and statin indication. Meta-analyses will be conducted using Comprehensive Meta-Analysis software (version 3.0, Biostat, New Jersey).

### Ethics and Dissemination

This study is based on published aggregate data. No identifiable individual data will be utilized, making a formal ethical approval unnecessary. This study has been registered with the International Prospective Register of Systematic Reviews (PROSPERO) with reference number CRD42016053191. If any aspect of the review is modified, this protocol will be updated in the registry. This systematic review will form a chapter of RO’s PhD thesis. The results of the study will also be disseminated through publications in peer-reviewed journals and presentations at relevant conferences and seminars.

## Results

The electronic database searches for relevant articles were completed in December 2016. The searches resulted in retrieval of over 10,000 articles. Removal of duplicates resulted in approximately 8000 articles that are currently being screened. The screening processes and analysis are expected to be completed by June 2017.

## Discussion

This systematic review, along with the potential to conduct meta-analyses, will provide important information regarding issues of adherence and persistence to statin therapy among older patients. The population of the world is aging [21], and most countries are expected to witness an expansion in the size of elderly populations. The elderly often face the greatest morbidity and mortality burden, making curative and preventive therapies intended to improve survival, minimize morbidity, and enhance quality of life extremely essential [50,51]. Enhancing adherence is one of the key ways of improving medication effectiveness. The findings of this study are therefore expected to inform the design of appropriate interventions that will improve adherence to statin therapy among elderly patients, so that optimal benefits can be accrued from such interventions.

### Strengths and Limitations of Study

The strengths and limitations of this study are summarized in Textbox 2.

**Study’s strengths and limitations.**

- Appropriate search strategy has been designed in consultation with an information management specialist who is experienced in conducting systematic reviews
- This study will be the first to evaluate statin adherence and persistence issues specific to older patients, and to compare variability across statin indications
- Our study does not impose any restriction on time period or geographic location
- Non-English articles will be excluded from the review, which may introduce some bias
- Study assessments will involve reviewer judgements, which may introduce bias

## Abbreviations

HMG-CoA: 3-hydroxymethyl-3-methylglutaryl coenzyme A
MPR: medicines possession ratio
NIH: National Institutes of Health
OR: odds ratio
PDC: proportion of days covered
PDT: proportion of doses taken
PICOS: population, intervention, comparison, outcome, and study design
PRISMA: Preferred Reporting Items for Systematic Reviews and Meta-Analyses
RCT: randomized controlled trial
